# Supplementary material for: A single pseudouridine on rRNA regulates ribosome structure and function in the mammalian parasite Trypanosoma brucei
Source: Nat Commun. 2023 Nov 20;14:7462. doi: 10.1038/s41467-023-43263-6 (PMC10662448; doi:10.1038/s41467-023-43263-6)
Supplement: Supplementary file 1 — Supplementary Information File [file 41467_2023_43263_MOESM1_ESM.pdf]

Supplementary Information Files

A single pseudouridine on rRNA regulates ribosome structure and function in the mammalian parasite *Trypanosoma brucei*

K. Shanmugha Rajan[1,2], Hava Madmoni[1], Anat Bashan[2], Masato Taoka[3], Saurav Aryal[1], Yuko Nobe[3], Tirza Doniger[1], Beathrice Galili[1], Amit Blumberg[3], Smadar Cohen-Chalamish[1], Schraga Schwartz[4], Andre Rivalta[2], Ella Zimmerman[2], Ron Unger[1], Toshiaki Isobe[3], Ada Yonath[2], and Shulamit Michaeli[1]

[1]The Mina and Everard Goodman Faculty of Life Sciences and Advanced and Nanotechnology Institute, Bar-Ilan University, Ramat-Gan 52900, Israel,
[2]Department of Chemical and Structural Biology, Weizmann Institute of Science, Rehovot 76100, Israel,
[3]Department of Chemistry, Graduate School of Science, Tokyo Metropolitan University, Minami-osawa 1-1, Hachioji-shi, Tokyo 192-0397, Japan,
[4]Department of Molecular Genetics, Weizmann Institute of Science, Rehovot 76100.

Inventory of Supplementary Information Files

Supplementary Figure 1. a) Validation of Ψ sites using direct RNA Oxford nanopore sequencing. b) Performance of Ψ site prediction based on T to C ratio from direct-RNA nanopore sequencing. c) Direct RNA nanopore sequencing detects developmentally regulated Ψ sites.

Supplementary Figure 2. (i) Localization of variable Ψ sites on the trypanosomatid ribosome. (ii) Localization of variable Ψ sites on the H69 domain.

Supplementary Figure 3. a) Integration of HDR template in TB11Cs6H1 sKO cells. b) Validation of TB11Cs6H1 sKO clones by Northern analysis.

Supplementary Figure 4. PCA of codon usage frequency in transcripts differentially expressed in TB11Cs6H1 sKO cells compared to PS.

Supplementary Figure 5. Time-dependent addback of TB11Cs6H1 recovers hnRNP F/H. a) (i) Addback of wild-type TB11Cs6H1 from rRNA locus. (ii) Growth of cells following wild-type TB11Cs6H1 addback from the rRNA locus. b) hnRNPF/H translation following wild-type snoRNA addback from the rRNA locus.

Supplementary Figure 6. sKO of TB7Cs1H1 snoRNA guiding Ψ522 on *T. brucei* LSU rRNA. a) Localization of Ψ sites on the H69 domain of *T. brucei* rRNA. b) CRISPR-Cas9 sKO of TB7Cs1H1. c) Growth of cells following TB7Cs1H1 sKO. d) hnRNPF/H translation is not affected in TB7Cs1H1 sKO.

Supplementary Figure 7. Purification and quantification of RP content in the 80S monosome.

Supplementary Figure 8. Flow chart presenting the cryo-EM data processing pipeline followed to reconstruct a cryo-EM map for PS and TB11CS6H1 sKO 80S ribosomes.

Supplementary Figure 9. a) Location of trypanosome-specific modifications in expansion segments (ES). b) Location of RNA modification in the focal points.

Supplementary Figure 10. Structural differences between PS and TB11Cs6H1 sKO 80S ribosome.

Supplementary Figure 11. Composition of BSF 80S monosomes.

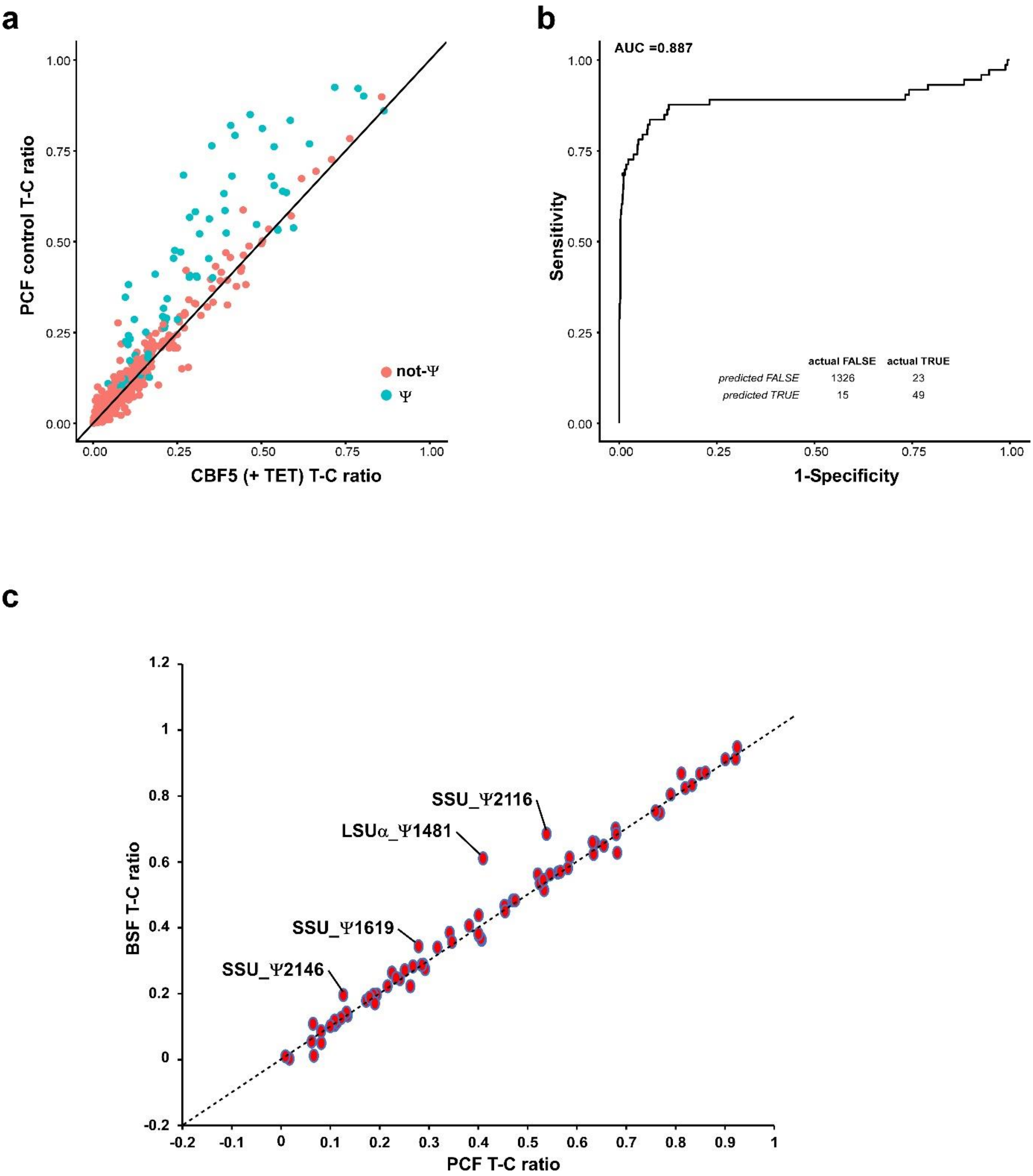

Supplementary Figure 1. a) Validation of Ψ sites using direct RNA Oxford nanopore sequencing. Total RNA derived from cells silenced for *cbf5* (+TET) and un-silenced PCF cells were subjected to nanopore sequencing. The mismatch ratio (T to C) of all uridine residues along the rRNA is shown. Data are presented as mismatch ratio of *cbf5* (+TET) along the x-axis, and control PCF cells along the y-axis. Ψ sites detected by Ψ-seq are indicated as blue dots and uridine residues are presented as orange dots. **b) Performance of Ψ site prediction based on T to C ratio from direct-RNA nanopore sequencing.** ROC curve (AUC=0.887) of the predicted Ψ sites is based on delta T to C ratio of PCF – CBF5+TET from nanopore sequencing. Confusion matrix of the optimal performance of ONT Ψ site prediction was generated using F1 score (optimal T to C delta =0.087). **c) Direct RNA nanopore sequencing detects developmentally regulated Ψ sites**. Data are presented as mutation ratio of BSF along the y-axis, and PCF cells along the x-axis. Only Ψ sites having FC>1.2 are shown.

i)

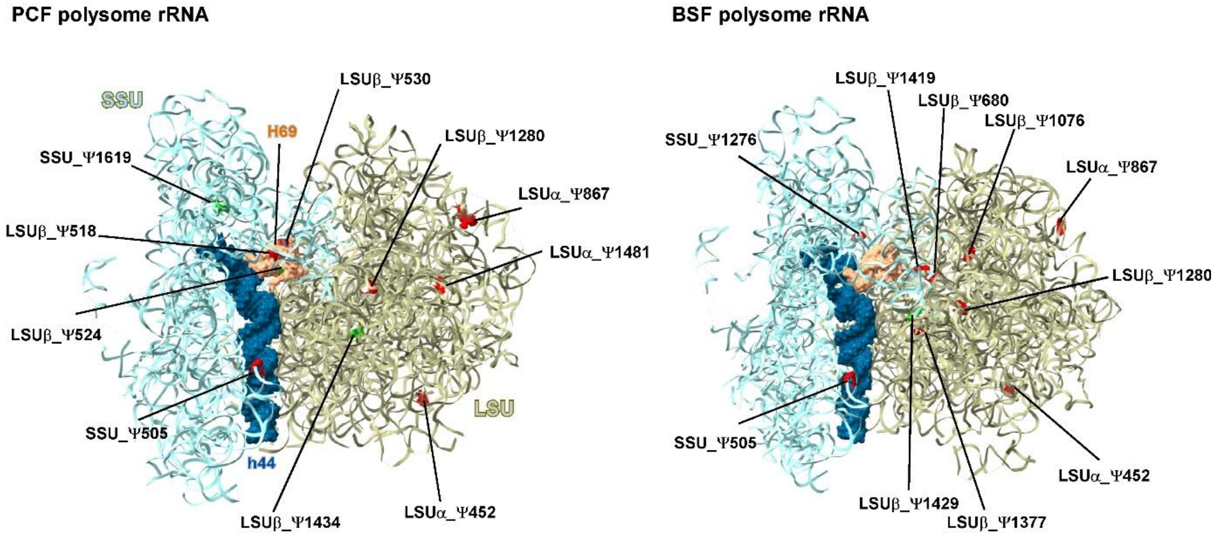

ii)

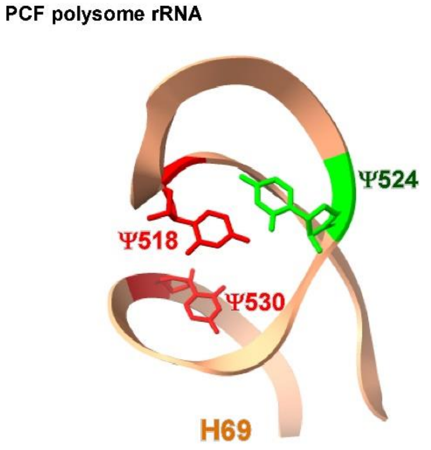

Supplementary Figure 2. (i) Localization of variable Ψ sites on the trypanosomatid ribosome. Ψ sites whose levels are increased in polysome (compared to total RNA) are colored red and those whose levels are decreased are colored green, respectively. The h44 and H69 domains are indicated. **(ii) Localization of variable Ψ sites on the H69 domain.**

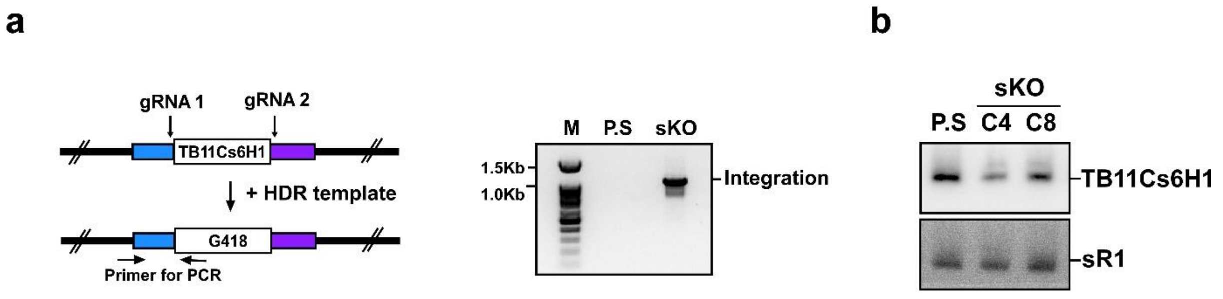

Supplementary Figure 3. a) Integration of HDR template in TB11Cs6H1 sKO cells. Schematic representation indicating the location of primers used to confirm proper integration of the HDR template. Gel-electrophoresis of the PCR product using the above-mentioned primers. The experiment was done one time. **b) Validation of TB11Cs6H1 sKO clones by Northern analysis.** Total RNA (10 µg) from PS and two clonal populations of KO was separated on a 10% denaturing polyacrylamide gel and detected by Northern blotting with complementary probes to the specified RNAs. SrRNA1 (sR1) RNA served as loading control. The experiment was done one time.

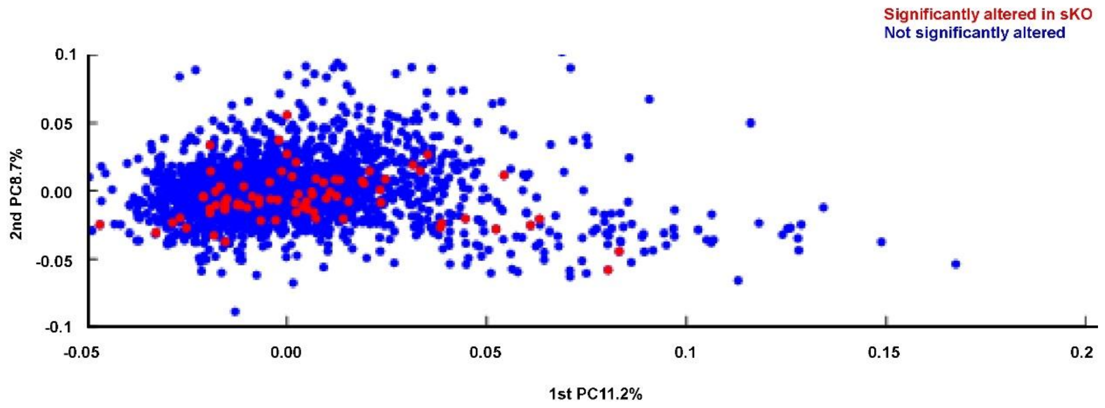

Supplementary Figure 4. PCA of codon usage frequency in transcripts differentially expressed in TB11Cs6H1 sKO cells compared to PS. Significantly altered proteins are indicated by red dots, and the remaining proteins by blue dots.

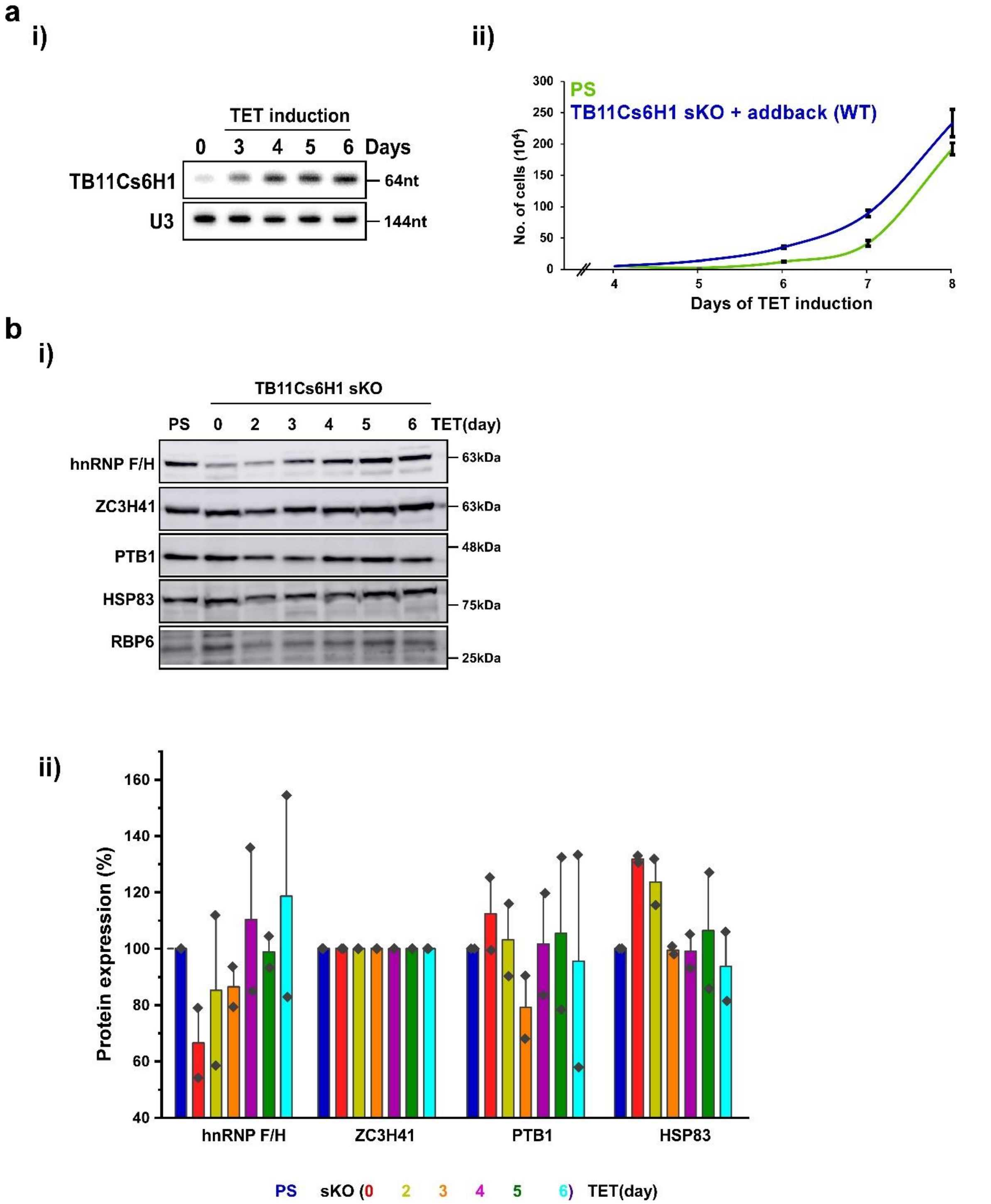

Supplementary Figure 5. Time-dependent addback of TB11Cs6H1 recovers hnRNP F/H. a) (i) Addback of wild-type TB11Cs6H1 from rRNA locus. The addback of TB11Cs6H1 snoRNA was confirmed by Northern analysis at the timepoints indicated. Total RNA (10 μg) from sKO cells induced for snoRNA expression (+TET) was separated on a 10% denaturing polyacrylamide gel and detected by Northern blotting with complementary probes to the specified snoRNAs. U3 RNA served as loading control. The experiment was done one time. **(ii) Growth of cells following wild-type TB11Cs6H1 addback from the rRNA locus.** The growth of PS and clonal population of sKO cells with snoRNA addback was compared at 27°C on the days indicated. Data are presented as mean ± S.E.M. Experiments were done in triplicate (n = 3). Each cell line was grown in three independent cultures, and growth was monitored in

parallel. **b) hnRNPF/H translation following wild-type snoRNA addback from the rRNA locus.** **(i)** Whole cell lysate from PS and TB11Cs6H1 sKO carrying wild-type snoRNA overexpression construct was subjected to western analysis with the indicated antibodies. The period of TET induction is indicated. The dilutions used for the antibodies were: hnRNPF/H (1:1,000), ZC3H41 (1:10,000), PTB1 (1:10,000), HSP83 (1:10,000), and RBP6 (1:1,000). **(ii)** Quantification of bands in (i) presented as mean ± S.E.M. Experiments were done in duplicate (n = 2). ZC3H41 served as the loading control. All samples shown in Figures bi were derived from the same experiment and blots were processed in parallel.

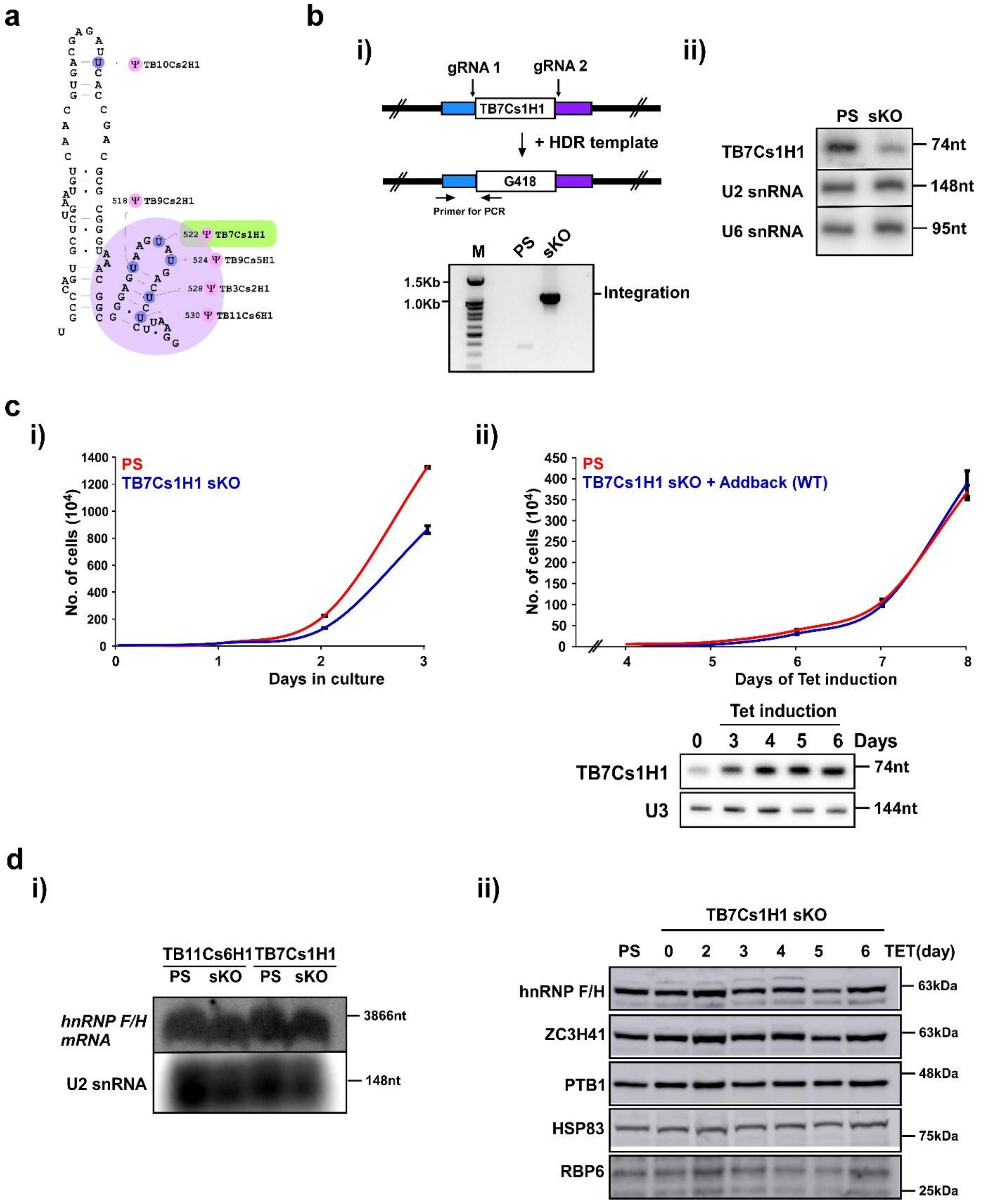

Supplementary Figure 6. sKO of TB7Cs1H1 snoRNA guiding Ψ522 on *T. brucei* LSU rRNA. a) Localization of Ψ sites on the H69 domain of *T. brucei* rRNA. The Ψ522 site guided by TB7Cs1H1 is highlighted in green. **b) CRISPR-Cas9 sKO of TB7Cs1H1. (i)** Schematic representation indicating the target of two guide RNAs and the site of HDR template integration. The integration of HDR template in TB7Cs1H1 snoRNA loci was confirmed by PCR using primers complementary to regions indicated by arrows. The experiment was done one time. **(ii) Validation of TB7Cs1H1 sKO by Northern analysis.** Total RNA (10 µg) from PS and clonal population of TB7Cs1H1 sKO was separated on a 10% denaturing polyacrylamide gel and detected by Northern blotting with probes complementary to the specified snoRNAs. The experiment was done one time. **c) Growth of cells following TB7Cs1H1 sKO. (i)** The growth of PS and the clonal population of TB7Cs1H1 sKO was compared at 27°C. Data are presented as mean ± S.E.M. Experiments were done in triplicate (n = 3). Each cell line was grown in three independent cultures and growth was monitored in parallel. **(ii)** Addback of TB7Cs1H1 from rRNA locus recovers cell growth. The addback of TB7Cs1H1 snoRNA was confirmed by Northern analysis during the period indicated. Total RNA (10 µg) from PS and a clonal population of sKO induced for snoRNA expression (+TET) was separated on a 10% denaturing polyacrylamide gel and detected by Northern blotting with probes complementary to the specified snoRNAs. **d) hnRNPF/H translation is not affected in TB7Cs1H1 sKO. (i) Northern analysis.** Total RNA (20 µg) from sKO and PS cells was separated on a 1.2% agarose/formaldehyde gel and detected by Northern blotting with complementary probes to the specified RNAs. U2 snRNA served as loading control. The experiment was done one time. **ii) Western analysis.** Whole cell lysate from PS, and TB7Cs1H1 sKO carrying wild-type TB7Cs1H1 snoRNA addback construct was subjected to western analysis with the indicated antibodies. The period of TET induction is indicated. The dilutions used for the antibodies were: hnRNPF/H (1:1,000), ZC3H41 (1:10,000), PTB1 (1:10,000), HSP83 (1:10,000), and RBP6 (1:1,000). ZC3H41, PTB1, HSP83 and MTAP serve as loading controls. The experiment was done one time. All samples shown in Figures bii and dii were derived from the same experiment and blots were processed in parallel.

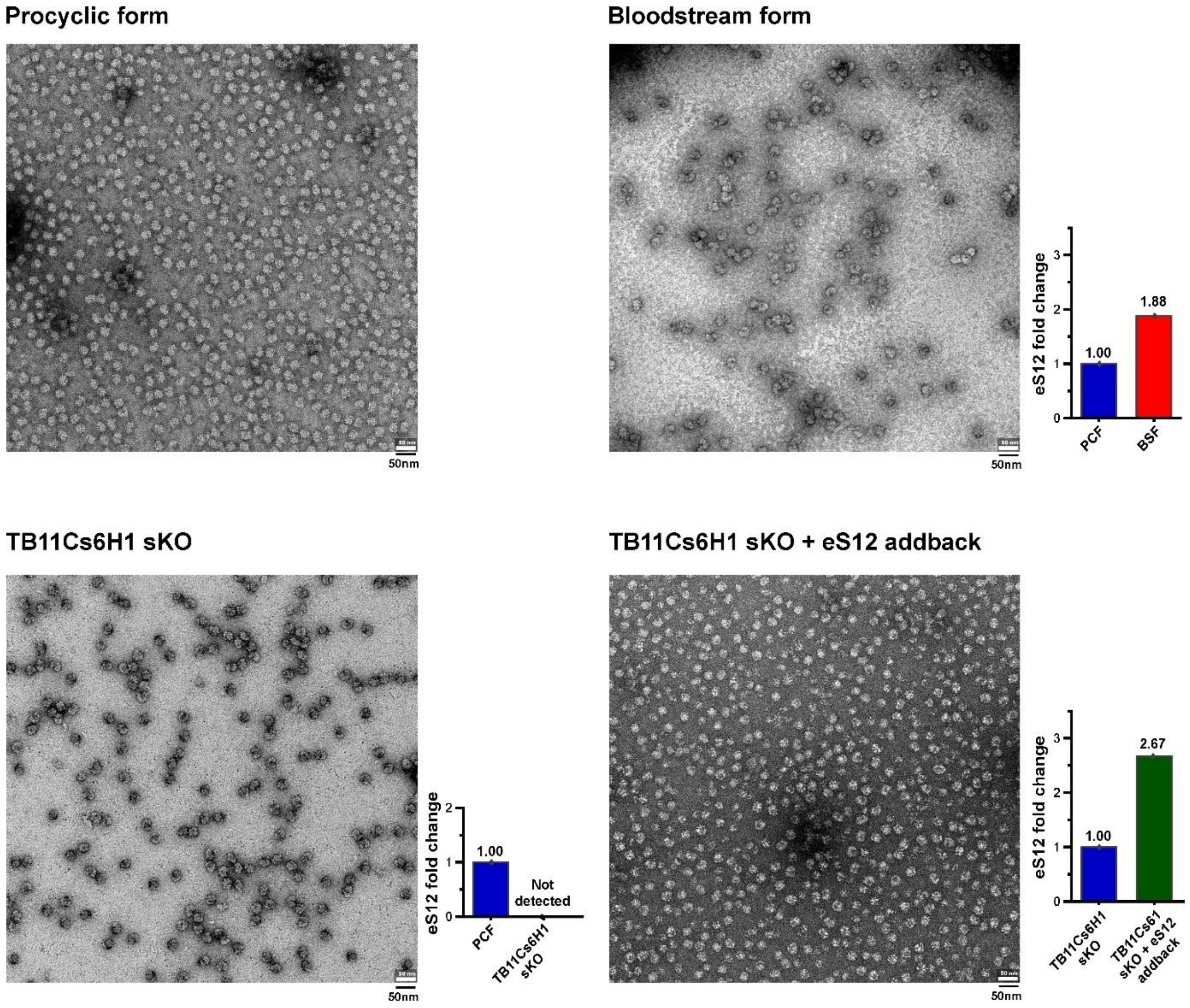

Supplementary Figure 7. Purification and quantification of RP content in the 80S monosome. 80S monosomes were purified as described in the Methods and subjected to negative staining with uranyl acetate. Representative TEM images are shown for each ribosome purification. The indicated ribosomes were labelled with dimethyl isotopes and subjected to reverse-phase chromatography and mass spectrometry. The quantification of eS12 compared to procyclic PS cells (PCF) is shown as a bar-graph. The experiment was done one time.

a. Parental strain 80S Ribosome

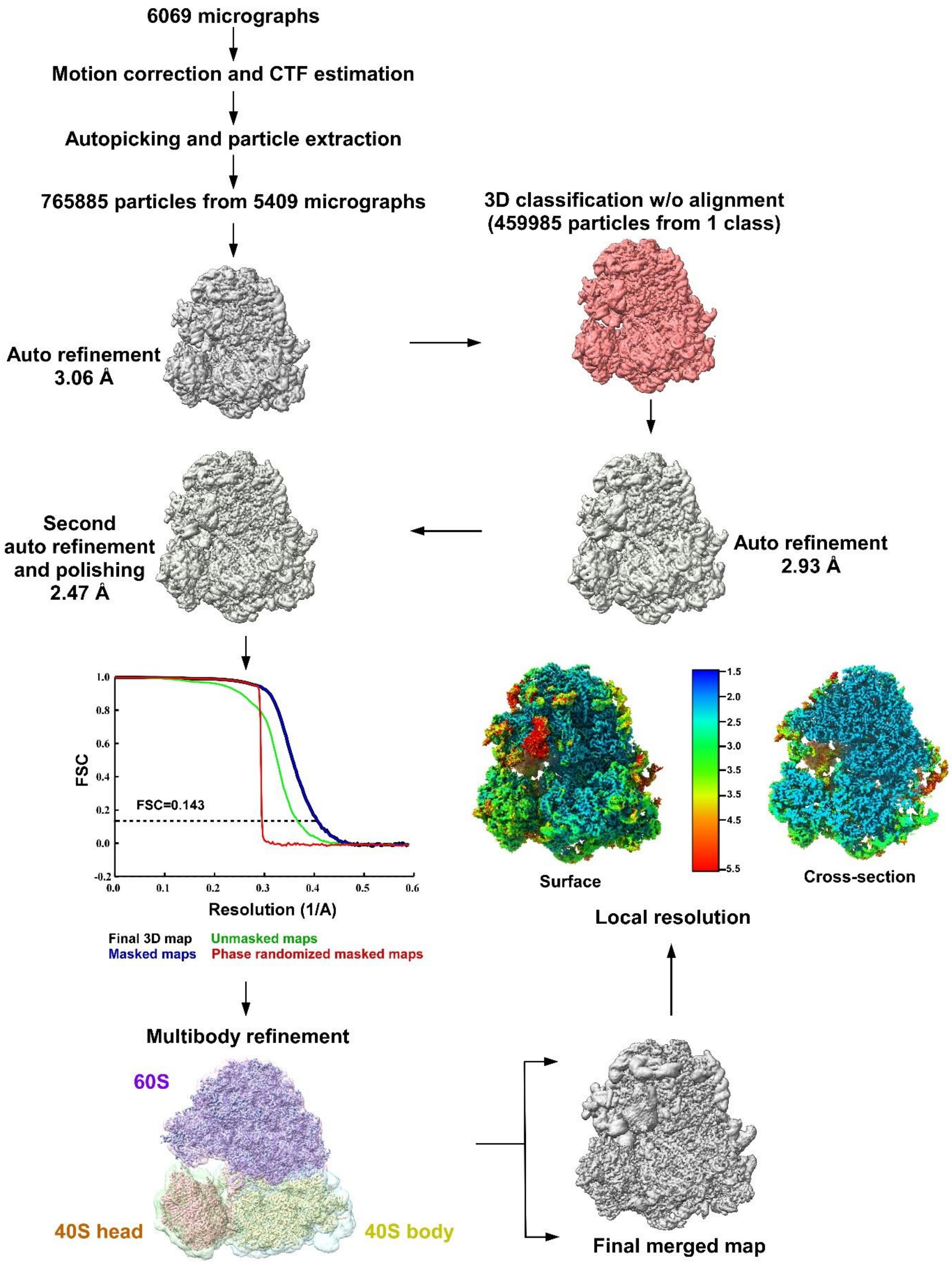

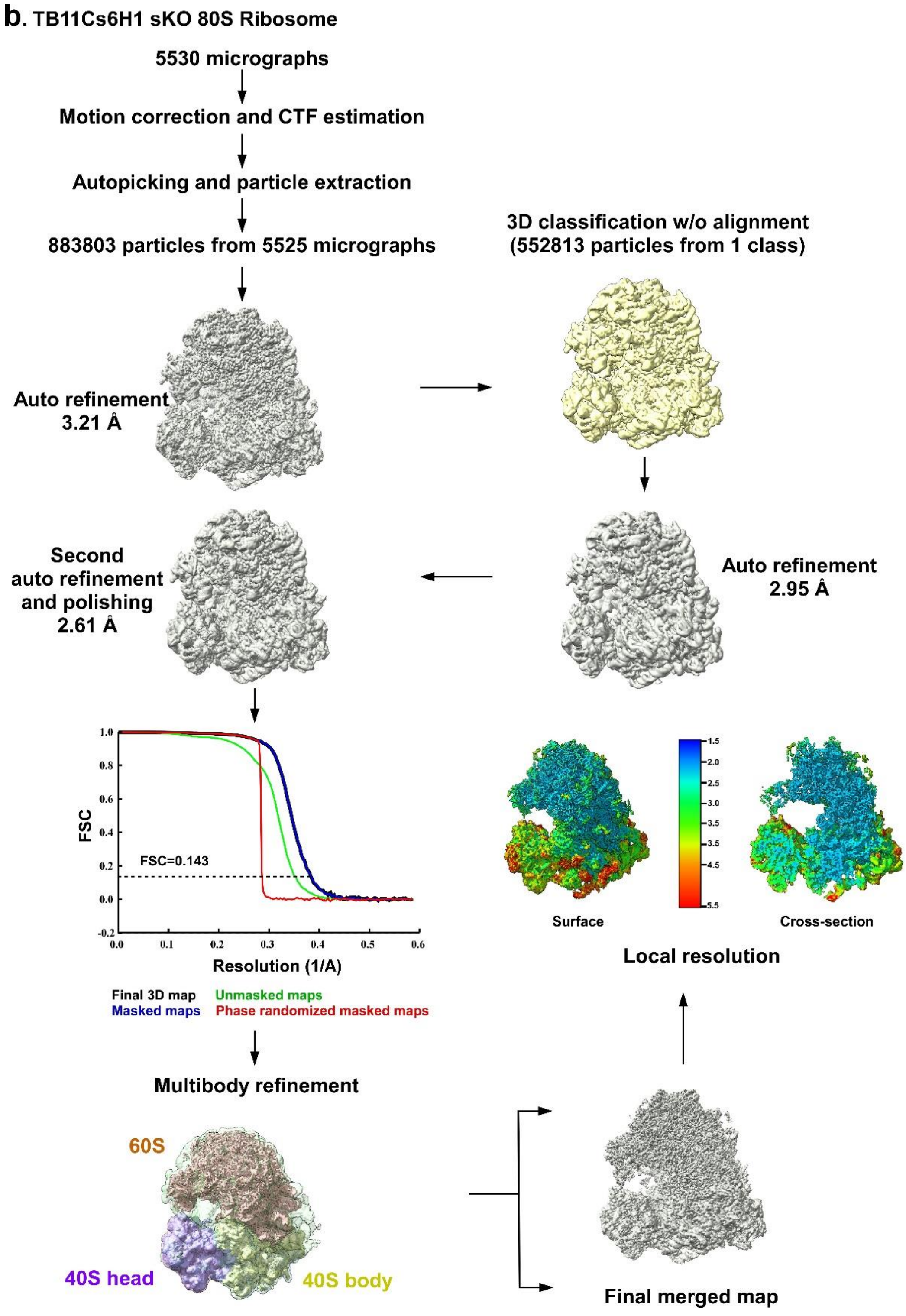

Supplementary Figure 8. Flow chart presenting the cryo-EM data processing pipeline followed to reconstruct a cryo-EM map for PS and TB11CS6H1 sKO 80S ribosomes. The number of micrographs, particles and resolution of corresponding maps are indicated. "Gold standard" FSC curves for the consensus EM map (black), unmasked map (green), masked map (blue), and phase randomized masked map (red) are presented. Surface rendering and cross-section of the cryo-EM density maps are colored according to local distribution of resolution. **a)** Parental strain. **b)** LM32Cs3H1 sKO.

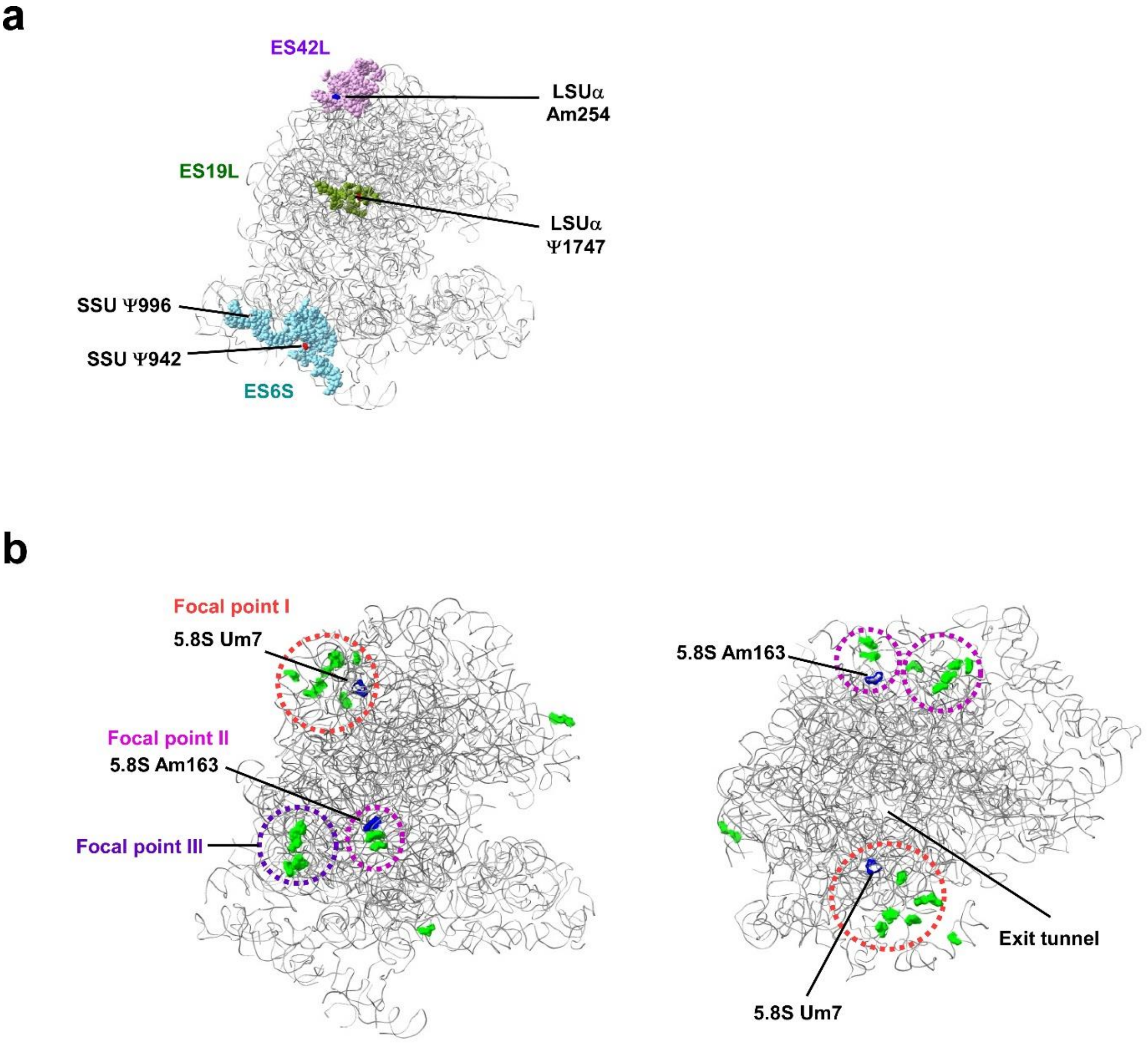

Supplementary Figure 9. a) Location of trypanosome-specific modifications in expansion segments (ES). The identities of rRNA modifications and ES are indicated. **b) Location of RNA modification in the focal points.** The identities of rRNA modifications and focal points are shown. The location of focal points is based on the published *L. donovani* cryo-EM structure [1].

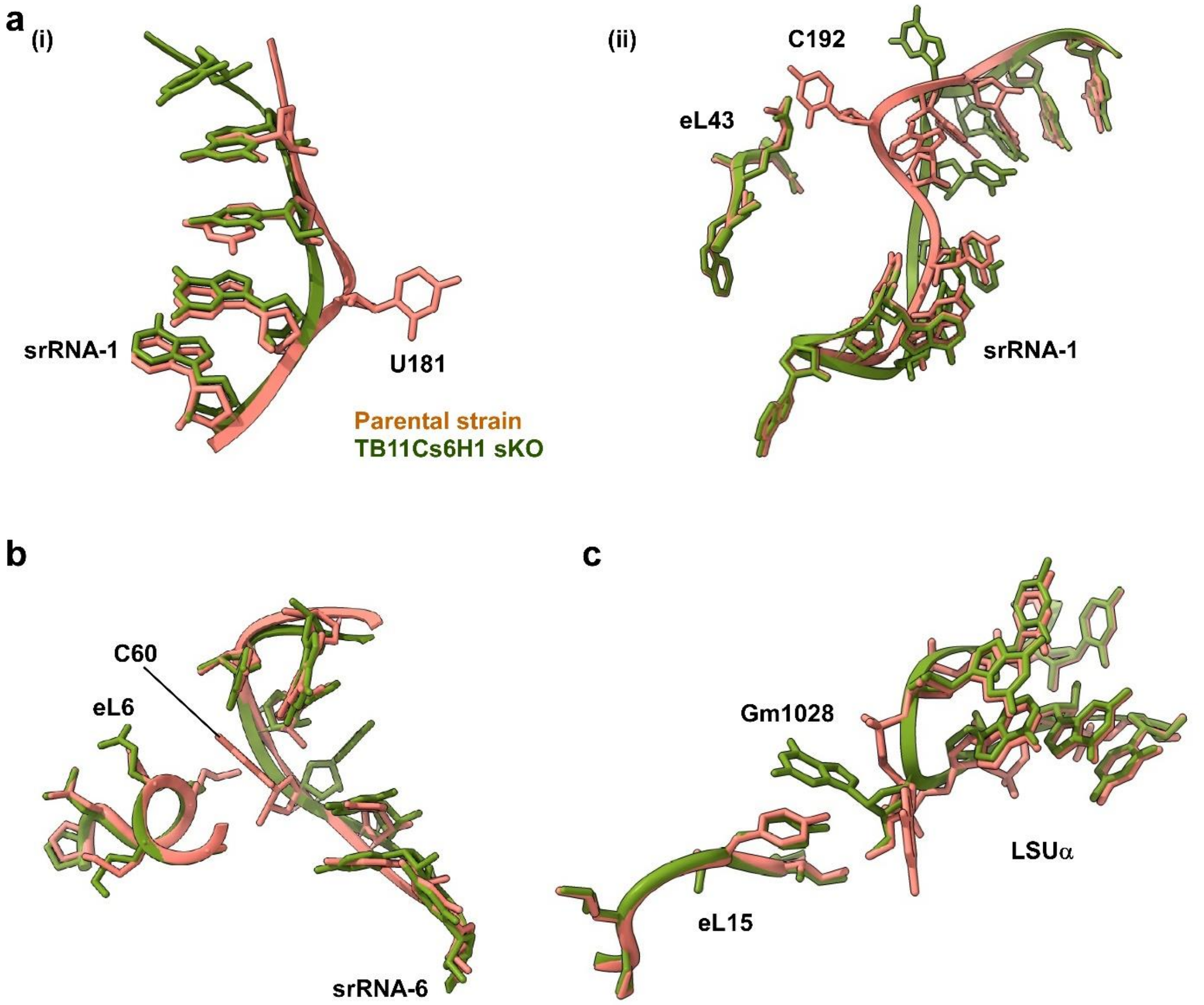

Supplementary Figure 10. Structural differences between PS and TB11Cs6H1 sKO 80S ribosome. Differences in srRNA-1 **(a)**, srRNA-6 **(b),** and LSUα (**c**) between PS and sKO ribosomes are shown in pink and green, respectively. The identity of rRNA, nucleotides and proteins are indicated.

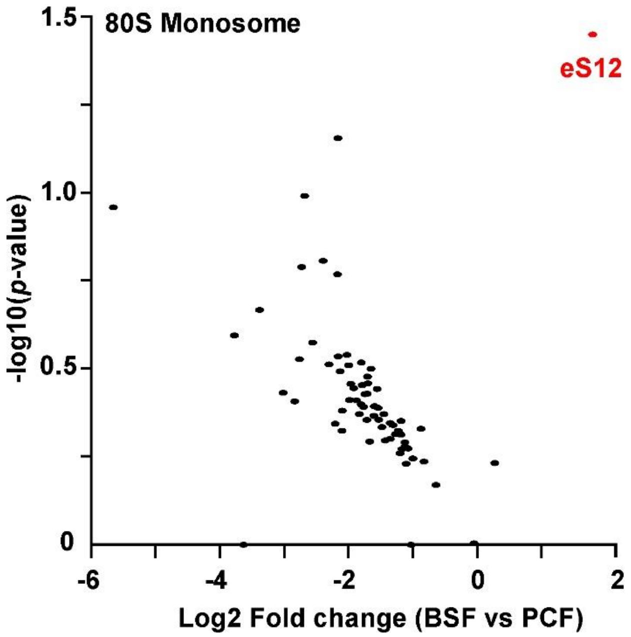

Supplementary Figure 11. Composition of BSF 80S monosomes. The 80S ribosome fraction was collected from the sucrose gradients, labelled with dimethyl isotopes and subjected to mass spectrometry as described in the Methods. Two biological replicates were used to calculate the fold-change (FC) in the two life stages of *T. brucei* (BSF and PCF). Data are presented as log2FC in the x-axis and log10(p-value) along the y-axis. Significant changes in protein abundance are indicated by red dots, and non-significant proteome changes in black.

SUPPLEMENTARY REFERENCES

1. Shalev-Benami, M. *et al.* Atomic resolution snapshot of Leishmania ribosome inhibition by the aminoglycoside paromomycin. *Nat. Commun.* **8**, 1589 (2017).
