# Supplementary material for: A single pseudouridine on rRNA regulates ribosome structure and function in the mammalian parasite Trypanosoma brucei
Source: Nat Commun. 2023 Nov 20;14:7462. doi: 10.1038/s41467-023-43263-6 (PMC10662448; doi:10.1038/s41467-023-43263-6)
Supplement: Supplementary file 3 — Description of Additional Supplementary Files [file 41467_2023_43263_MOESM3_ESM.pdf]

Description of Additional Supplementary Files

File Name: Supplementary Data 1
Description: **Validation of Ψ sites detected by Ψ-seq using HydraPsiSeq.** The stoichiometry of Ψ at each nt is represented as a PsiScore. Ψ sites not detected by HydraPsiSeq are highlighted in blue, and Ψ sites that were not reduced upon CBF5 depletion are highlighted in yellow.

File Name: Supplementary Data 2
Description: **Complete stoichiometry of Ψ on *T. brucei* rRNA calculated by HydraPsiSeq for each Ψ site.** Data are presented as mean+/-S.E.M. A minimum of three independent replicates were used to calculate the PsiScore. Fold change (FC) >1.2 is highlighted in orange, and FC <1.2 is highlighted in green. *p*-value was determined by Student's t-test Two-tailed distribution. No adjustments were made for multiple comparisons.

File Name: Supplementary Data 3
Description: **Comparative analysis of Ψ detected by Ψ-seq, HydraPsiSeq, and nanopore sequencing.** The mutation ratio (T-C) for each Ψ site detected by nanopore sequencing is presented. Hypermodified Ψ sites detected in both Ψ-seq and HydraPsiSeq are indicated. Ψ sites not reduced upon CBF5 depletion are highlighted in orange.

File Name: Supplementary Data 4
Description: **Complete stoichiometry of Ψ on *T. brucei* rRNA calculated by tandem LC-MS.** Fold change (FC) >1.2 is highlighted in orange, and FC <1.2 is highlighted in green. The comparison between other Ψ mapping methods is presented. *p*-value for HydraPsiSeq was determined by Student's t-test Two-tailed distribution. No adjustments were made for multiple comparisons.

File Name: Supplementary Data 5
Description: **Quantitative proteome of TB11Cs6H1 sKO cells. (**i) Whole cell lysates were labelled with dimethyl isotopes and subjected to reversed-phase chromatography and mass spectrometry. Three biological replicates were used to calculate the fold-change (FC) following sKO. *p*-value was determined by one-sample t-test. Benjamini-Hochberg correction for multiple hypothesis testing (SignificanceB) was performed. The identity of significantly altered proteins, their nomenclature, FC compared to parental cells (P.S), and the p-values are indicated. (ii) Raw data of all three replicates used for proteome quantification. (iii) RNA-seq of PS and sKO pol(A) RNA. Corrected *p*-value and fold-change was calculated using DESeq2. The *p*-values were corrected for multiple testing using the Benjamini and Hochberg method.

File Name: Supplementary Data 6
Description: **Features of proteins affected in TB11Cs6H1 sKO cells.** The identity of significantly altered proteins, their nomenclature, fold change (FC) compared to parental cells (PS), and the *p* value are indicated. The length of UTRs was derived from both TriTrypDB (https://tritrypdb.org/) and Kolev *et al.*[80]. The proteome data from the two life stages of the parasite was extracted from Urbaniak *et al.,* [39].

File Name: Supplementary Data 7
Description: **Quantitative proteome of TB11Cs6H1 sKO and BSF 80S monosome.** Ribosomes purified by sucrose gradient were labelled with dimethyl isotopes and subjected to reversed-phase chromatography and mass spectrometry. Two biological replicates were used to calculate the fold-change (FC) following sKO. *p*-value was determined by one-sample t-test. Benjamini-Hochberg correction for multiple hypothesis testing (SignificanceB) was performed. The identity of proteins, their nomenclature, FC compared to procyclic PS, and the *p*-values are provided.

File Name: Supplementary Data 8
Description: **eS12 addback rescue expression of specific proteins.** Whole cell lysates from TB11Cs6H1 sKO cells overexpressing MYC-eS12 or empty vector were labelled with dimethyl isotopes and subjected to mass spectrometry. Identity of proteins, their nomenclature, and fold change compared to sKO cells expressing empty vector. Proteins whose expression level was recovered following eS12 addback are indicated in orange. Only one biological replicate was used to calculate the fold-change (FC) following sKO. *p*-value was determined by one-sample t-test. Benjamini-Hochberg correction for multiple hypothesis testing (SignificanceB) was performed.

File Name: Supplementary Data 9
Description: **Summary of regions modelled in *T. brucei* 80S ribosome in this study.** Description of the protein, residues modelled, sequence, and PDB chain ID.

File Name: Supplementary Data 10
Description: **List of all RNA modifications modelled in *T. brucei* 80S ribosome in this study.** Nucleotide identity, type of RNA modification, and potential snoRNA to guide each modification.

File Name: Supplementary Data 11
Description: **Complete stoichiometry of base-methylation and Nm on *T. brucei* rRNA calculated by tandem LC-MS.** Nucleotide identity, type of RNA modification, stoichiometry, and putative RNA fragments detected.

File Name: Supplementary Data 12
Description: **List of primers used in this study.**

File Name: Supplementary Data 13
Description: **Summary of samples deposited in PRIDE database.** The identity of samples labelled with heavy and light dimethyl isotopes and their description.
