# Supplementary material for: A single pseudouridine on rRNA regulates ribosome structure and function in the mammalian parasite Trypanosoma brucei
Source: Nat Commun. 2023 Nov 20;14:7462. doi: 10.1038/s41467-023-43263-6 (PMC10662448; doi:10.1038/s41467-023-43263-6)
Supplement: Supplementary file 17 — Reporting Summary [file 41467_2023_43263_MOESM17_ESM.pdf]

nature portfolio

Corresponding author(s): SHULAMIT MICHAELI

Last updated by author(s): Oct 20, 2023

Reporting Summary

Nature Portfolio wishes to improve the reproducibility of the work that we publish. This form provides structure for consistency and transparency in reporting. For further information on Nature Portfolio policies, see our Editorial Policies and the Editorial Policy Checklist.

Statistics

For all statistical analyses, confirm that the following items are present in the figure legend, table legend, main text, or Methods section.

n/a	Confirmed	
☐	☒	The exact sample size (*n*) for each experimental group/condition, given as a discrete number and unit of measurement
☒	☐	A statement on whether measurements were taken from distinct samples or whether the same sample was measured repeatedly
☐	☒	The statistical test(s) used AND whether they are one- or two-sided<br>*Only common tests should be described solely by name; describe more complex techniques in the Methods section.*
☒	☐	A description of all covariates tested
☐	☒	A description of any assumptions or corrections, such as tests of normality and adjustment for multiple comparisons
☐	☒	A full description of the statistical parameters including central tendency (e.g. means) or other basic estimates (e.g. regression coefficient) AND variation (e.g. standard deviation) or associated estimates of uncertainty (e.g. confidence intervals)
☐	☒	For null hypothesis testing, the test statistic (e.g. *F*, *t*, *r*) with confidence intervals, effect sizes, degrees of freedom and *P* value noted<br>*Give P values as exact values whenever suitable.*
☒	☐	For Bayesian analysis, information on the choice of priors and Markov chain Monte Carlo settings
☒	☐	For hierarchical and complex designs, identification of the appropriate level for tests and full reporting of outcomes
☒	☐	Estimates of effect sizes (e.g. Cohen's *d*, Pearson's *r*), indicating how they were calculated

Our web collection on statistics for biologists contains articles on many of the points above.

Software and code

Policy information about availability of computer code

Data collection

A Titan Krios electron microscope (Thermo Fischer Scientific) operating at 300 kV equipped with K3 direct electron detector (Gatan Inc.) was used for collecting cryo-EM micrographs at liquid nitrogen temperature at a nominal magnification of 105 000x, with a pixel size of 0.85 Å/pixel and a dose rate of ~1 electron/Å2/s. Defocus values ranged from −0.5 to −1.5 µm.
Northern blots were imaged using GE Typhoon FLA 9500.
Western blots were imaged using Amersham Imager 680.
Ethidium stained agarose gel was imaged using BioRad Gel Doc XR+ Gel Documentation System.

Data analysis

The paired end reads obtained from each sample were aligned to the Trypanosoma brucei ribosomal RNA using Smalt v_0.7.5 (http://www.sanger.ac.uk/resources/software/SMALT/) with default parameters. For each sample, the resulting bam file was sorted and filtered for proper pairs using SAMtools v1.9 and then converted to a BED file using the bamtobed module from the BEDtools v2.26.0 Suite. Using an in-house Perl script on each bed file, the number of reads whose 5'-end alignments initiate at that base for each position on the rRNA was calculated. The total coverage for each base was calculated using genomecov module from the BEDtools v2.26.0 Suite. These files were then used as input for the R scripts (https://github.com/FlorianPichot/HydraPsiSeqPipeline).

The RNA-sequencing reads were aligned to the T. brucei genome (v5) using Smalt v_0.7.5 (http://www.sanger.ac.uk/resources/software/SMALT/) with default parameters. After alignment, the expression level of mRNA was quantified using HTSeq-count to count the number of reads aligned to each gene in the TriTrypDB T. brucei gtf file (https://tritrypdb.org). The gene count tables were used as input for the R-Bioconductor package DESeq2 (v1.38.3) to perform differential gene expression analysis using the default parameters. mRNAs with an absolute log2 fold-change of >/=1 and FDR < 0.05 were considered differentially expressed.

Demultiplexing of the barcoded direct RNA sequencing libraries was performed using DeePlexiCon with stringent criteria of 0.9. Reads were base-called with guppy_basecaller using the following parameters: -r -c rna_r9.4.1_70bps.cfg. Reads were then mapped to the T. brucei reference genome by minimap2 (-ax map-ont) and transformed to BAM files, sorted and indexed using SAMtools.

The mass spectrometry data were analyzed using the Proteome Discoverer 1.4 (Thermo) software, searching against the T. brucei from the TriTrypDB v.35 database (https://tritrypdb.org). Results were filtered with rank 1 peptides and 1% false discovery rate. The ratios were normalized according to the protein's median ratio. Perseus software (https://maxquant.net/perseus/) was used for statistical analysis of the data. Significant outliers relative to a given population were calculated using intensity-dependent calculation. The truncation was based on the Benjamini-Hochberg correction for multiple hypothesis testing (SignificnceB) 60. For combined analysis of different replicates, one-sample t-test was used to determine if the mean was significantly different from a fixed value (0).

Relion 3.1 was used for data processing 61. Motion correction and contrast transfer function parameters were estimated using Motioncor2 and CTFFIND-3, respectively. The extracted particles were subjected to several rounds of unsupervised 3D classification using a low-pass filtered cryo-EM density map. 3D classes similar to 80S particles were selected and subjected to auto-refinement in Relion. Following initial refinement, particles were subjected to CTF refinement, Bayesian polishing, and refinement. The resulting high-resolution 3D density map was then subjected to a cycle of multibody refinement using separate masks for the large subunit (LSU), the head, and body regions of SSU. The gold standard Fourier shell correlation (FSC) value criterion of 0.143 was used for determining averaged map resolutions as implemented in Relion 3.1. Local resolution was estimated using Resmap.

The Mg2+, Zn2+, Na+, and K+ ion compositions were modeled according to the recently described criteria. Model refinement was performed using an iterative approach, including real-space refinement and geometry regularization in COOT followed by real-space refinement using the PHENIX Real_space_ refine tool. The final model was validated using MolProbity .

Bioinformatics scripts used in this study are available at https://github.com/michaelilab/Tb_rRNA_pseudo_translation.

For manuscripts utilizing custom algorithms or software that are central to the research but not yet described in published literature, software must be made available to editors and reviewers. We strongly encourage code deposition in a community repository (e.g. GitHub). See the Nature Portfolio guidelines for submitting code & software for further information.

Data

Policy information about availability of data

All manuscripts must include a data availability statement. This statement should provide the following information, where applicable:

- Accession codes, unique identifiers, or web links for publicly available datasets
- A description of any restrictions on data availability
- For clinical datasets or third party data, please ensure that the statement adheres to our policy

The RNA sequencing data generated in this study have been deposited in the NCBI BioProject database under the accession number PRJNA79183 (https://www.ncbi.nlm.nih.gov/bioproject/PRJNA791832). The mass spectrometry proteomics data were deposited to the ProteomeXchange Consortium via the PRIDE partner repository with the dataset identifier PXD030544. The data can be accessed using the following details, Username: reviewer_pxd030544@ebi.ac.uk, Password: U9zvOAMk. The cryo-EM density maps of the T. brucei 80S ribosome have been deposited in the Electron Microscopy Data Bank (EMDB) under accession numbers EMD-17208 and EMD-17212. Atomic coordinates and structure factors have been deposited in the Protein Data Bank (PDB) under accession codes 8OVA and 8OVE.

Research involving human participants, their data, or biological material

Policy information about studies with human participants or human data. See also policy information about sex, gender (identity/presentation), and sexual orientation and race, ethnicity and racism.

Reporting on sex and gender	N/A
Reporting on race, ethnicity, or other socially relevant groupings	N/A
Population characteristics	N/A
Recruitment	N/A
Ethics oversight	N/A

Note that full information on the approval of the study protocol must also be provided in the manuscript.

Field-specific reporting

Please select the one below that is the best fit for your research. If you are not sure, read the appropriate sections before making your selection.

☒ Life sciences ☐ Behavioural & social sciences ☐ Ecological, evolutionary & environmental sciences

For a reference copy of the document with all sections, see nature.com/documents/nr-reporting-summary-flat.pdf

Life sciences study design

All studies must disclose on these points even when the disclosure is negative.

Sample size	We choose sufficient sample sizes to determine whether the outcome is statistically significant. For all main experiments we performed at least three biologically independant replicates to perform statistical analysis. For validation certain validation experiments only one replicate was performed to support other experiments.
Data exclusions	No data was excluded
Replication	At least three biological independent replicate was performed for most experiments. All attempts for replication were successful.
Randomization	All cell lines were randomly assigned to the experimental and control groups.
Blinding	All experiments were not blinded.

Reporting for specific materials, systems and methods

We require information from authors about some types of materials, experimental systems and methods used in many studies. Here, indicate whether each material, system or method listed is relevant to your study. If you are not sure if a list item applies to your research, read the appropriate section before selecting a response.

Materials & experimental systems

n/a	Involved in the study	
☐	☒	Antibodies
☐	☒	Eukaryotic cell lines
☒	☐	Palaeontology and archaeology
☒	☐	Animals and other organisms
☒	☐	Clinical data
☒	☐	Dual use research of concern
☒	☐	Plants

Methods

n/a	Involved in the study	
☒	☐	ChIP-seq
☐	☒	Flow cytometry
☒	☐	MRI-based neuroimaging

Antibodies

Antibodies used	HA antibody was purchased from BioLegend and its catalog number is 16B12. The antibody was validated by the manufacturer.<br>MYC antibody was purchased from Santa-cruz and its catalog number is 9E10. The antibody was validated by the manufacturer.<br>hnRNPF/H, MTAP, PTB1, PTB2, U2AF35 and ZC3H41 was prepared in Prof. Shulamit Michaeli lab. All these antibody was validated in previous publications from the lab.<br>RBP10 antibody was prepared in Prof. Christine Clayton lab. The antibody was validated in previous publications from the lab.<br>RBP6 antibody was prepared in Prof. Christian Tschudi lab. The antibody was validated in previous publications from the lab.<br>The dilution used are, hnRNPF/H (1:1,000), MTAP (1:10,000), PTB1 (1:10,000), PTB2 (1:10,000),U2AF35 (1:10,000), HSP83 (1:10,000), ZC3H41 (1:10,000), RBP6 (1:1,000) and RBP10 (1:1,000).
Validation	HA antibody was was validated by the manufacturer, BioLegend.<br>MYC antibody was validated by the manufacturer, Santa-cruz.<br>hnRNPF/H, MTAP, PTB1, PTB2, U2AF35 and ZC3H41 was validated by Prof. Shulamit Michaeli lab in previous publications from the lab.<br>RBP10 antibody was validated by Prof. Christine Clayton lab in previous publications from the lab.<br>RBP6 antibody was validated by the Prof. Christian Tschudi lab in previous publications from the lab.

Eukaryotic cell lines

Policy information about cell lines and Sex and Gender in Research

Cell line source(s)	Procyclic form (PCF) T. brucei, strain 29-13, which carries integrated genes for the T7 polymerase and the tetracycline repressor, was grown in SDM-79 medium supplemented with 10% fetal calf serum, in the presence of 50 µg/ml hygromycin. Cells were grown in the presence of 15 µg/ml G418 for generating the RNAi silenced cell lines. PCF 1313 strain (a gift from C. Clayton, ZMBH, Heidelberg, Germany) which carries the tetracycline repressor gene used for expressing the Cas9 protein was grown in SDM-79 medium supplemented with 10% fetal calf serum, in the presence of phleomycin. The bloodstream form (BSF) of T. brucei 427 (cell line 1313-514) was aerobically cultivated at 37oC under 5% CO2 in HMI-9 medium supplemented with 10% fetal calf serum, 2ug/ml G418, and 2.4 ig/ml phleomycin.
Authentication	None of the cell lines were authenticated.

Mycoplasma contamination | Cell lines were not tested for Mycoplasma contamination

Commonly misidentified lines (See ICLAC register) | No commonly misidentified cell lines were used

Flow Cytometry

Plots

Confirm that:

- [x] The axis labels state the marker and fluorochrome used (e.g. CD4-FITC).
- [ ] The axis scales are clearly visible. Include numbers along axes only for bottom left plot of group (a 'group' is an analysis of identical markers).
- [ ] All plots are contour plots with outliers or pseudocolor plots.
- [ ] A numerical value for number of cells or percentage (with statistics) is provided.

Methodology

Sample preparation | Procyclic cells were washed in methionine free (phenol-red free) SDM-79 media, treated with 50 µM Click-iT® AHA (L-azidohomoalanine) reagent (Invitrogen) in methionine-free (phenol-red free) SDM-79 media, and incubated for 30 minutes at 27oC according to the manufacturer's protocol. The cells were fixed using 1.6% formaldehyde, and permeabilized with Triton:Tween 20 (1:0.1%) in 1xPBS. The cells were incubated with Click-iT® reaction cocktail for 30 minutes, and washed once with 3% BSA in 1xPBS. The incorporated Click-iT® AHA was determined by FACS Aria using the Alexa 488 channel.

Instrument | FACS Aria

Software | Data were collected by BD ARIA software and analysed by FlowJo (www.flowjo.com)

Cell population abundance | 10000 cell were measured for each experiment

Gating strategy | N/A

- [ ] Tick this box to confirm that a figure exemplifying the gating strategy is provided in the Supplementary Information.
